# Do microsaccades track shifting but not sustaining covert attention?

**DOI:** 10.1073/pnas.2309431120

**Published:** 2023-08-21

**Authors:** Freek van Ede

**Affiliations:** ^a^Institute for Brain and Behavior Amsterdam, Department of Experimental and Applied Psychology, Vrije Universiteit Amsterdam, Amsterdam 1081 BT, The Netherlands

Microsaccades—small fixational eye movements—have been shown to parallel the direction of covert shifts of attention (1, 2), consistent with a key role of the brain’s oculomotor system in orchestrating covert attention (3). Accordingly, microsaccades have been proposed to be a useful marker of covert attention (4, 5), with translational promise such as for tracking disorders of attention in psychological conditions or with aging.

A new study by Willet & Mayo (6) casts shadow on this prospect (see also ref. 7), showing that microsaccades do not track currently attended visual objects. The authors cued which of two visual objects was more likely to contain a sudden change in orientation (the target) that would appear after a variable delay after object onset. Cues were presented prior to each block of ~50 trials. Across two different protocols, the authors report weak or no biasing of microsaccade direction to the attended visual object, despite clear attentional facilitation in performance and neural activity. Hence, the authors conclude, using microsaccades as a marker for attention is problematic.

**Fig. 1.**
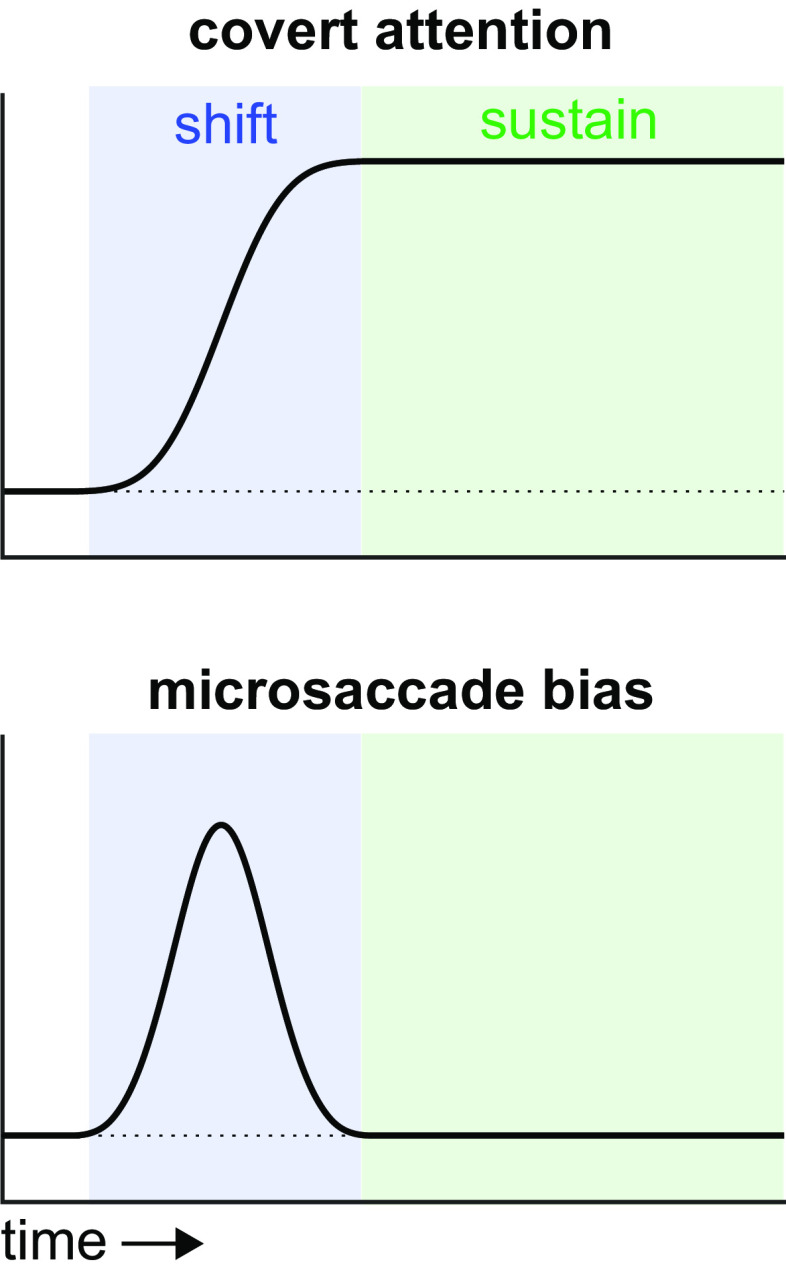
Hypothetical scenario where microsaccades predominantly track shifting but not sustaining covert selective attention.

Attention, however, is not a unitary construct or state. It comprises multiple functional stages, with a relevant distinction being between shifting and sustaining attention (8, 9). Could it be that microsaccades—and, by extension, upstream oculomotor brain circuitry—predominantly correlate with specific attentional stages, such as shifting (as hypothetically illustrated in Fig. 1)? Indeed, prior studies linking microsaccade direction to covert attention (e.g., refs. 1, 2, and 10) employed tasks that promoted attentional shifting, while Willet & Mayo’s task required sustaining attention to the visual object that had been cued before the block.

Microsaccade biases when shifting attention among visual contents in working memory (5, 10) provide a case in point. Directing attention inside working memory engages shifting attention to specific internal representations, but also sustaining priority of attended representations for upcoming behavior. In this context, despite such sustained demands, my colleagues and I recently observed transient biasing of fixational gaze behavior (figure 2 in ref. 5), consistent with the proposed role in shifting but not sustaining attention.

When putting the hypothesis that microsaccades predominantly track shifting but not sustaining covert attention to the test, it will be vital to consider two points. First, the separation by shifting vs. sustaining is unlikely to be all-or-none. During sustained task periods, attention may lapse and require reshifting to attended objects [possibly accounting for the residual microsaccade bias in Willet & Mayo (6)]. Second, even if microsaccades predominantly track shifting, this does not imply that all attention shifts will necessarily be paralleled by microsaccades. The relation between shifting covert attention and microsaccades is still likely to be probabilistic such that only when microsaccades happen to occur during attentional shifts, microsaccade direction and latency will correlate with the shifting process (10).

As Willet & Mayo conclude, we must be careful when linking microsaccades to covert attention. I fully agree. Carefully delineating what stages of attention are paralleled in microsaccades, as well as in neural activity in upstream oculomotor brain areas, are important questions awaiting research.
